# Influence of Disease Duration on Circulating Levels of miRNAs in Children and Adolescents with New Onset Type 1 Diabetes

**DOI:** 10.3390/ncrna4040035

**Published:** 2018-11-21

**Authors:** Nasim Samandari, Aashiq H. Mirza, Simranjeet Kaur, Philip Hougaard, Lotte B. Nielsen, Siri Fredheim, Henrik B. Mortensen, Flemming Pociot

**Affiliations:** 1 Copenhagen Diabetes Research Centre (CPH-DIRECT), Department of Paediatrics, Herlev and Gentofte Hospitals, Herlev Ringvej 75, 2730 Herlev, Denmark; nasim.samandari@regionh.dk (N.S.); aah2003@med.cornell.edu (A.H.M.); simranjeet.kaur@regionh.dk (S.K.); lottebn2@gmail.com (L.B.N.); sirifredheim@dadlnet.dk (S.F.); henrik.bindesboel.mortensen@regionh.dk (H.B.M.); 2 Steno Diabetes Center Copenhagen, 2820 Gentofte, Denmark; 3 Research Unit of Epidemiology, Biostatistics and Biodemography, University of Southern Denmark, 5230 Odense M, Denmark; phhougaard@mail.dk; 4 Faculty of Health and Medical Sciences, University of Copenhagen, 2200 Copenhagen N, Denmark

**Keywords:** children, immunology, miRNA, partial remission phase, type 1 diabetes

## Abstract

Circulating microRNAs (miRNAs) have been implicated in several pathologies including type 1 diabetes. In the present study, we aimed to identify circulating miRNAs affected by disease duration in children with recent onset type 1 diabetes. Forty children and adolescents from the Danish Remission Phase Cohort were followed with blood samples drawn at 1, 3, 6, 12, and 60 months after diagnosis. Pancreatic autoantibodies were measured at each visit. Cytokines were measured only the first year. miRNA expression profiling was performed by RT-qPCR. The effect of disease duration was analyzed by mixed models for repeated measurements adjusted for sex and age. Eight miRNAs (hsa-miR-10b-5p, hsa-miR-17-5p, hsa-miR-30e-5p, hsa-miR-93-5p, hsa-miR-99a-5p, hsa-miR-125b-5p, hsa-miR-423-3p, and hsa-miR-497-5p) were found to significantly change in expression (adjusted *p*-value < 0.05) with disease progression. Three pancreatic autoantibodies, ICA, IA-2A, and GAD65A, and four cytokines, IL-4, IL-10, IL-21, and IL-22, were associated with the miRNAs at different time points. Pathway analysis revealed associations with various immune-mediated signaling pathways. Eight miRNAs that were involved in immunological pathways changed expression levels during the first five years after diagnosis and were associated with variations in cytokine and pancreatic antibodies, suggesting a possible effect on the immunological processes in the early phase of the disease.

## 1. Introduction

There is an increased interest in epigenetic mechanisms to understand disease pathogenesis and progression. Epigenetics involves the heritable modifications of gene expression without changes in the genome sequence, leading to an altered, sometimes unfavorable, phenotype, e.g., resulting in metabolic diseases such as diabetes. While it is recognized that a genetic component is essential to the risk of developing type 1 diabetes (T1D), data from twin studies suggests that genetics alone cannot explain the disease development [1,2]. Furthermore, the increasing incidence of T1D in younger children is at a rate which is too fast to be only accounted for by a genetic change. Thus, the role of other pathogenic influences, such as diet, environment, viral infections, interferons, and epigenetic modifications, has been suggested to contribute to the development of autoimmune diabetes [3,4,5,6,7]. 

MicroRNAs (miRNAs) are small non-coding RNAs with potent post-transcriptional regulatory properties which have shown potential as biomarkers for different pathophysiologies [8]. During recent years, the promising features of miRNAs as biomarkers associated with insulin production, residual β-cell function, and disease complications of diabetes have been investigated [9,10,11]. The knowledge emerging on the role of epigenetic control, such as miRNAs, DNA methylation patterns, and histone modifications, indicates that these regulatory mechanisms have an important function in T1D [12,13,14]. 

An important stage in the natural history of T1D is the spontaneous remission phase, which can be either complete, with temporary insulin independence, or partial. During this phase, which can last from weeks to several months, a decreased requirement of exogenous insulin is experienced and an improved metabolic control is observed [15]. The partial remission (PR) phase is a potential temporal opening for investigating the important immunoregulatory events taking place when the remaining β-cells are still able to produce enough insulin. Currently, the strategies employed so far have not yet been able to unravel the underlying mechanisms during this phase, making the development of future immunotherapies with the capabilities to extend the PR challenging.

Previously, we identified six miRNAs predictive for residual β-cell function and metabolic control in children newly diagnosed with T1D [11]. In the present study, we hypothesized that circulating miRNAs reflect the ongoing autoimmune process during the first year after disease onset. Thus, the objective of this study was to identify circulating miRNAs, which hold the potential to serve as biomarkers for the autoimmune and immunological status in newly diagnosed children and adolescents with T1D. 

## 2. Results

### 2.1. Technical Controls Prior to miRNA Profiling

The demographic and anthropometric data characterizing the study cohort is presented in Table 1 and has also recently been described elsewhere [11]. Plasma samples from a subset of children and adolescents (*n* = 40) from this cohort collected at different time-points (1, 3, 6, 12, and 60 months) after diagnosis were subjected to miRNA profiling. The steady expression levels of the RNA spike-ins, added in the RNA isolation and complementary DNA (cDNA) synthesis steps, indicated that the processes of RNA extraction, reverse transcription, and qPCR were efficient, and no inhibitors were present in the samples. Hemolysis of the samples varied between 5 and −2, thus indicating that the samples were not affected by hemolysis 

### 2.2. miRNA Quantification in 182 Plasma Samples

A selected panel of 179 human serum/plasma miRNAs was analyzed over the five time points after diagnosis. An average of 163 miRNAs were quantified and 100 were quantified in all samples. 

### 2.3. Effect of Disease Duration on miRNA Expression Levels during the First Five Years after Diagnosis

**Figure 1:**
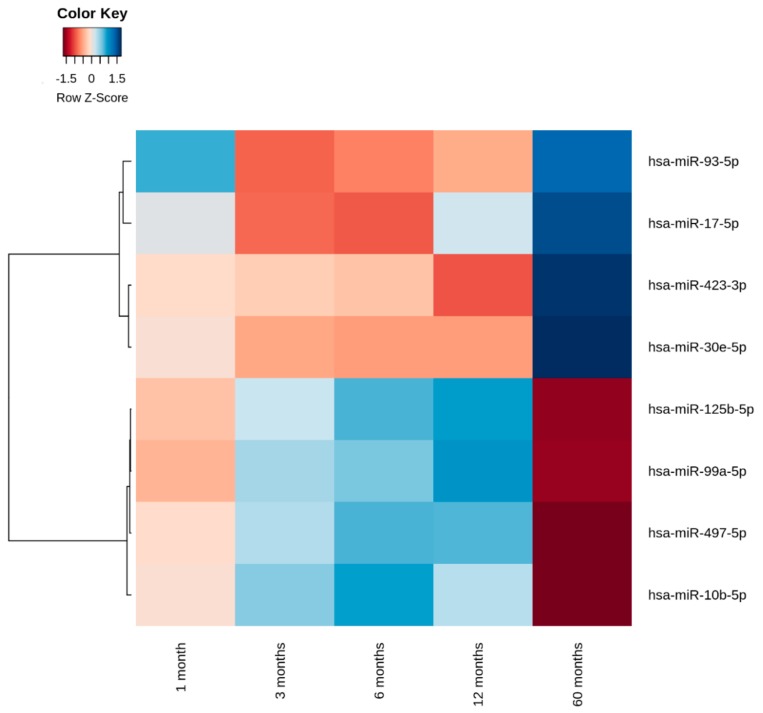
The eight candidate microRNAs (miRNAs) with changing expression levels during the first five years after diagnosis. The normalized expression (∆Cp) values of the miRNAs at different time-points are clustered based on the distance measure (which is calculated as 1-cor) and transformed into Z-scores. cor = Pearson’s correlation coefficient.

Analysis by mixed model for repeated measurements indicated that 28 miRNAs varied in expression levels during the first five years after diagnosis (*p* < 0.05). After correcting for multiple testing, eight miRNAs (hsa-miR-10b-5p, hsa-miR-17-5p, hsa-miR-30e-5p, hsa-miR-93-5p, hsa-miR-99a-5p, hsa-miR-125b-5p, hsa-miR-423-3p, and hsa-miR-497-5p) showed variations in expression levels during the first five years after disease onset (*p* < 0.05) (Table 2). The results of these eight candidate miRNAs were adjusted for age and sex. After adjustments, the data revealed that age only had an effect on hsa-miR-423-3p (*p* = 0.0088, data not shown). Neither HbA1c nor stimulated C-peptide (SCP) correlated with any of the quantified miRNAs. The average expression levels of the eight candidate miRNAs with altered expression levels during the first five years are shown as a heat map in Figure 1.

The normalized expression (global mean method and adjusted for age and sex) levels (∆Cp) of hsa-miR-17-5p, hsa-miR-30e-5p, hsa-miR-93-5p, and hsa-miR-423-3p declined within the first 12 months after diagnosis and subsequently increased from one to five years after diagnosis. By contrast, the levels of hsa-miR-10b-5p, hsa-miR-99a-5p, hsa-miR-125b-5p, and hsa-miR-497-5p increased during the first 12 months and subsequently decreased between one and five years after diagnosis. Five miRNAs (hsa-miR-10b-5p, hsa-miR-17-5p, hsa-miR-99a-5p, hsa-miR-125b-5p, and hsa-miR-497-5p) were expressed with a lower ∆Cp value compared to the average of all samples at each time point, and the remaining three miRNAs (hsa-miR-30e-5p, hsa-miR-93-5p, and hsa-miR-423-3p) were more abundantly expressed compared to the average of all samples at all time-points (Figure 1 and Table S1).

### 2.4. Identification of miRNA Target Genes

The target genes for the eight candidate miRNAs were retrieved from two sources: miRTarBase [16] and TargetScan [17] using cyTargetLinker [18]. In total, 996 target genes for eight miRNAs as predicted by both tools were used for further analysis (Table S1). The pathway enrichment analysis was performed using PantherDB [19]. The pathway analysis revealed enrichment of various signaling pathways including epidermal growth factor (EGF), fibroblast growth factor (FGF), integrin, platelet-derived growth factor (PDGF), transforming growth factor (TGF) β, and apoptosis signaling pathways, as shown in Table 3. The detailed pathway analysis including the enriched target genes associated with each pathway are listed (Table S2).

### 2.5. Partial remission and Immunological Status

Using the new definition for PR with IDAA1c ≤ 9 in the study population of 40 children and adolescents, 19 (51%) children were in PR 3 months after diagnosis, and 4 (12%) and 1 (3%) were in PR after 12 and 60 months, respectively. 

Of the five autoantibodies tested, seroconversion was most profound for ZnT8tripleAb during the study period. Insulin antibodies (IA) increased from 80 to 100% during the first year after diagnosis (Table 1) and remained positive in all the children between three and twelve months in the study population and the study cohort. Seroconversion for IA was observed in twenty-two percent of the children five years after diagnosis (Table 1). The Spearman correlation analysis performed on the association of the eight candidate miRNAs and the anti-islet autoantibodies measurements showed that three of the candidate miRNAs were statistically significantly (*p* < 0.036) associated with the measured autoantibodies before correcting for multiple testing. Islet-cell autoantibodies (ICA) were negatively associated with hsa-miR-99a-5p at 3 months after diagnosis. At the same time point, ICA and IA-2A were negatively associated with hsa-miR-125b-5p. Finally, GADA65 was positively associated with hsa-miR-17-5p 6 months after diagnosis (Table 4). 

### 2.6. Association of the Eight Candidate miRNAs to Cytokines 

The Spearman correlation analysis performed between the eight candidate miRNAs and 35 measured cytokines at 1, 3, 6, and 12 months after diagnosis showed, before correction for multiple testing, that hsa-miR-10b-5p correlated negatively with IL-21 (*r* = −0.63, *p* = 0.0051) at 3 months, whereas hsa-miR-17-5p at 1 month correlated positively with IL-21 (*r* = 0.62, *p* = 0.0048). Likewise, hsa-miR-17-5p was positively associated with IL-10 and IL-4 at 1 month (*r* = 0.68, *p* = 0.0015; *r* = 0.70, *p* = 0.0008; respectively). Finally, hsa-miR-30e-5p correlated positively with IL-22 at 6 months (*r* = 0.71, *p* = 0.0015).

## 3. Discussion

Aberrantly circulating miRNA levels in plasma, serum, and urine samples from children with T1D were assessed recently by Osipova et al. [20], and the number of studies exploring the prognostic potential of miRNAs in different settings is steadily increasing [21,22,23]. Our group recently showed, in the same cohort as the one used in present study, that six miRNAs predicted stimulated C-peptide, HbA1c, and the insulin dose adjusted HbA1c (IDAA1c) the first year after disease onset [11]. In the present study, we identified miRNAs with expression levels that were influenced by disease progression, but not associated to the decline in β-cell function. In this prospective study, disease progression had a statistically significant effect on the expression levels of eight candidate miRNAs, irrespective of age and sex. Further bioinformatics evaluation of the validated targets of these miRNAs and the observed cytokine and pancreatic antibody associations support a possible immunological impact reflected by circulating miRNAs during the PR. However, cytokine profiles were only quantified in about half of the study participants in the Danish Remission Phase Cohort due to technical reasons. Thus, the study was under-powered for fully exploring these potential associations. The pathway-based analysis identified highly significant pathways associated with the target genes of eight miRNA candidates. The top highly significant pathways included angiogenesis, TGF-β, cholecystokinin receptor (CCKR), EGF, PDGF, apoptosis, FGF, integrin signaling, and the Ras pathway. *STAT3*, a potential target gene for hsa-miR-93-5p and hsa-miR-125b-5p, is associated with angiogenesis, the Ras pathway, EGF receptor signaling, CCKR signaling, and PDGF signaling pathways. *MTOR* (a serine/threonine protein kinase), which is a target gene for hsa-miR-99a-5p, is associated with the PDGF signaling pathway. MTOR is a central regulator of cellular metabolism, growth, and survival [24]. The activation of MTOR regulates various effector functions of CD4+ and CD8+ T cells, and MTOR inhibitors (i.e., rapamycin) are used as potential immunosuppressive therapeutics for organ transplants, metabolic diseases, and various autoimmune disorders [25]. Furthermore, MTOR has been demonstrated to be involved in the onset and progression of autoimmune disorders including diabetes [26]. *PIK3CD*, a target gene for hsa-miR-10b-5p is a known mediator of immune responses and is associated with several pathways including angiogenesis, apoptosis signaling, EGF receptor signaling, PDGF signaling, FGF signaling, integrin signaling, and the Ras pathway. 

One of the strengths of this study was the phenotypically thorough characterization of the study participants who were followed with repeated measurements initially after diagnosis and during the first five years thereafter. The present study also provides data from pathway analysis together with cytokine and pancreatic antibody variations associated with miRNAs during PR to suggest that miRNAs regulating genes that control immune recognition could be involved in the autoimmune processes during the initial progression of the disease. In our previous study [11], six miRNAs analyzed in the same population could predict residual β-cell function and glycemic control (SCP, HbA1c, and IDAA1c) in children with new onset T1D the first five years after diagnosis. Accumulating evidence supports that miRNAs play a crucial role in the regulation of pancreatic β-cell function under normal and pathophysiological conditions, such as insulin biosynthesis, insulin exocytosis, and β-cell expansion, and may have clinical significance in diabetes [11,27]. The current study further provides data, in the same study population, on different miRNAs, which change expression levels during the first five years after diagnosis and have a possible involvement in immunological pathways. Three pancreatic autoantibodies, ICA, IA-2A, and GADA65, and four cytokines, IL-4, IL-10, IL-21, and IL-22, were associated with the miRNAs at different time points. Interestingly, hsa-miR-17-5p correlated with interleukin-4 and interleukin-10 levels, which have anti-inflammatory effects and dampens the autoimmune process. However, these stratifications were under-powered due to limited sample sizes and the limited number of study participants who were followed five years after diabetes diagnosis. This decreased the statistical power to conclude on the potential role of the eight candidate miRNAs during the PR. The observed variability of the candidate miRNAs need further validation in larger cohorts, and their immunological function in autoimmune diabetes also needs to be elucidated in further detail.

In conclusion, this study identified eight circulating miRNAs that changed in expression levels, irrespective of age and sex, during the first five years after diabetes diagnosis, which have the potential to be used as biomarkers for the immunological progression and definition of the optimal state for immune intervention during the initial phase of T1D.

## 4. Materials and Methods

### 4.1. Study Population and Samples

The Danish Remission Phase Cohort comprised 129 children (66 boys) with T1D. The study was a longitudinal multicenter investigation conducted in four pediatric out-patient clinics with enrolment during the years 2004 to 2005. Plasma samples were drawn on fasting state after a meal stimulation test at 1, 3, 6, 12, and 60 months after diagnosis and stored in a bio-bank at −80 °C until further use. Diabetes diagnosis was classified according to the World Health Organization criteria [28]. The reasons for drop-outs from the cohort or loss of follow up at the 5-year visit were either adolescents transferred to adult clinics, changed residences, or unwillingness to participate. The exclusion criteria were suspicions of other types of diabetes (maturity-onset diabetes of the young (MODY), type 2 diabetes, or secondary diabetes) and/or if patients were initially treated outside the center for more than five days. The 40 participants who completed the follow-up at five years diabetes duration were slightly younger with a lower body mass index (BMI) and had lower postprandial C-peptide levels 12 months after diagnosis compared to the full cohort, but they were similar in terms of sex distribution, HbA1c, and hormone levels. The study was performed according to the criteria of the Helsinki II Declaration and was approved by the Danish National Committee on Biomedical Research Ethics (Journal number: H-KA-04010-m). Older children gave their assent and all parents or guardians gave written informed consent.

### 4.2. Partial Remission (PR) Phase

Different definitions for the PR phase have been suggested. Previously, it was defined as HbA1c near or within the normal range and a daily insulin dose requirement of <0.5 units/kg/day [29]. A new definition combining both values has recently been proposed: insulin dose-adjusted HbA1c, and IDAA1c, which suggests that a value ≤9 indicates partial remission [30,31]. This definition has been recommended by the International Society for Pediatric and Adolescent Diabetes (ISPAD) [32].

### 4.3. Pancreatic Anti-Islet Autoantibodies

Islet autoantibody measurements were measured in serum samples from all included children in the Danish Remission Phase Cohort. Titers of the anti-islet autoantibodies (insulin antibodies (IA), insulinoma associated antigen-2 autoantibody (IA-2A), autoantibodies against glutamic acid decarboxylase (GAD65A), and islet-cell autoantibodies (ICA)) were analyzed at 1, 3, 6, 12, and 60 months after diagnosis according to the methods described earlier [33]. The zinc transporter 8 variant containing all three isotypes arginine, tryptophan, and glutamine (ZnT8RWQ or ZnT8tripleAb) were analyzed by a so called triple mix radioligand binding assay (RBA) as previously described [34,35] at the time points 1, 3, 6, and 12 months after diagnosis. Another method was used for analyzing the "triple mix" ZnT8 autoantibodies (ZnT8tripleAb) 60 months after diagnosis [36].

The cut-off values for anti-islet autoantibody positivity were 5.36 relative units (RU) for GAD65A, 0.77 RU for IA-2A, 2.5 Juvenile Diabetes Foundation units (JDFU) for ICA, and 2.80 RU for IA. The cut-off limit for the triple mix assay, used the first year after diagnosis, was 58 U/mL, and for the assay by Salonen et al. [36] used five years after diagnosis, the cut-off value was set to 0.61 RU.

### 4.4. Cytokine Measurements

Cytokine status was measured for half of the recruited study participants in the Danish Remission Phase Cohort at 1, 3, 6, and 12 months after diagnosis. Cytokine measurements were performed using an in-house-developed and -validated multiplex immunoassay (Laboratory of Translational Immunology, University Medical Center, Utrecht, The Netherlands) based on Luminex technology (xMAP, Luminex, Austin TX, USA) [37]. 

### 4.5. miRNA Expression Profiling and Normalization Method 

miRNAs were quantified from a total of 182 plasma samples drawn from the subgroup of 40 children and adolescents who participated in the 5-year visit. RNA extraction was performed using the miRCURYTM RNA Isolation Kit-Biofluids and RT-qPCR was performed with ExiLENT SYBR^®^ Green Master Mix according to the manufacturer’s instructions (Exiqon A/S, Vedbaek, Denmark). All RT-qPCR assays were performed in a LightCycler480^®^ Real-Time PCR System in 384 well plates (Roche, Hvidovre, Denmark). Quality control was done on a subset of samples (*n* = 10) with synthetic spike-in RNAs as a quality control of the RNA isolation and cDNA synthesis. A detailed description of the RNA isolation and quality control procedure is outlined in [9]. Assessment of hemolysis was evaluated by comparing the levels of hsa-miR-451a (miRNA highly expressed in erythrocytes) with hsa-miR-23a-3p (stable in plasma/serum and not affected by hemolysis) as described by Blondal et al. [38]. 

Each plasma sample was run on a selected assay containing a panel of 179 predefined human serum/plasma miRNAs (V3; Exiqon), including miRNAs known to be involved in immunological and metabolic diseases. The 100 miRNAs with complete data that were quantified in all samples in the miRNA expression profiling were employed for the normalization of data using the global mean method, since this approach has been shown to be the most stable normalizer according to Mestdagh et al. [39]. The formula used to calculate the normalized Cp values was:

ΔCp = average Cp (all samples) − assay Cp (sample)

### 4.6. Validation of Target Genes and Pathway Analysis

The validated and predicted target genes for the eight candidate miRNAs found in this study were retrieved from two sources: miRTarBase [16] (experimentally verified miRNA interactions retrieved from automated text mining in PubMed) and TargetScan [17] (predicted miRNA interactions) using the cyTargetLinker app in CytoScape [18]. The pathway-based enrichment analysis was performed using PantherDB [19]. The *p*-values were calculated using the binomial test and adjusted for multiple corrections using the Bonferroni method. 

### 4.7. Statistical Analysis

All statistical analyses were performed using SAS version 9.4 (SAS Institute, Cary, NC, USA). The miRNA levels with complete data were included for analysis and were managed on a base-2 logarithmic scale (log2). The influence of diabetes duration on circulating levels of miRNAs were analyzed by a linear mixed model for repeated measurements including the covariates for sex and age at the visit (to account for the potential age development) and with an unstructured covariance matrix. *p*-Values for the differentially expressed miRNAs associated with disease duration were further adjusted according to the Hochberg approach (*p* < 0.05). For antibodies and cytokines, the Spearman correlation analysis used the measured values and included values below the detection limit as a value below all others; it was performed to determine the relationship between variables and was considered significant with a corresponding *p* ≤ 0.05.

## Figures and Tables

**Table 1:** Characteristics of 40 children and adolescents (study population) from the Danish Remission Phase Cohort and the entire cohort, respectively.

Study Population	1 Month	3 Months	6 Months	12 Months	60 Months
*n* (girls/*n*)	40 (24)	37 (22)	33 (19)	34 (20)	38 (23)
Age at diagnosis (years)	8.7 (3.4)	-	-	-	-
HbA1c (DCCT, %)	9.1 (1.3)	7.3 (1.1)	7.8 (1.6)	7.9 (1.1)	8.4 (1.0)
HbA1c (IFCC,mmol/mol)	76 (14.2)	56 (12.0)	62 (17.5)	63 (12.0)	68 (11.0)
C-peptide (pmol/L)	629 (371)	528 (323)	436 (333)	257 (251)	62 (76)
IDAA1c	10.8 (2.0)	9.2 (1.5)	10.1 (2.2)	11.0 (1.8)	12.0 (1.9)
Insulin dose (units/kg/24 h)	0.45 (0.27)	0.47 (0.23)	0.58 (0.26)	0.78 (0.27)	0.88 (0.32)
Autoantibody positivity (%)					
GAD65A	55	62	61	56	55
IA	80	100	100	100	78
IA-2A	73	68	70	68	74
ICA	93	92	94	94	92
ZnT8tripleAb	65	70	75	76	26

Data are presented as means (SD), unless otherwise stated. SCP: stimulated C-peptide. IDAA1c = HbA1c (percent) + [4 × insulin dose (units per kilogram per 24 h)]. GAD65A: autoantibodies against glutamic acid decarboxylase (GAD). IA: insulin autoantibodies. IA-2A: islet antigen 2 antibody. ICA: islet-cell antibody. ZnT8tribleAb: Zinc transporter 8 triple mix antibody variant containing the isotypes glutamine, arginine, and tryptophan. Missing data are either due to no visit, missing or lost sample, or drop-out.

**Table 2:** Effect of disease duration on miRNA expression level.

miRNA	Unadjusted *p*-Value	Adjusted *p*-Value
hsa-miR-99a-5p	<0.00001	0.00022
hsa-miR-30e-5p	<0.00001	0.00027
hsa-miR-497-5p	<0.00001	0.00041
hsa-miR-10b-5p	0.00001	0.00072
hsa-miR-423-3p	0.00001	0.00131
hsa-miR-125b-5p	0.00003	0.00252
hsa-miR-17-5p	0.00011	0.01057
hsa-miR-93-5p	0.00012	0.01094
hsa-miR-146a-5p	0.00094	0.08678
hsa-miR-484	0.00195	0.17716
hsa-miR-185-5p	0.00208	0.18726
hsa-miR-24-3	0.00291	0.25943
hsa-miR-660-5p	0.00944	0.83059
hsa-miR-25-3p	0.01088	0.94673
hsa-let-7b-5p	0.01133	0.97420
hsa-miR-320a	0.01236	0.98546
hsa-miR-223-3p	0.01248	0.98546
hsa-let-7g-5p	0.01553	0.98546
hsa-miR-20a-5p	0.01815	0.98546
hsa-let-7i-5p	0.02298	0.98546
hsa-miR-142-3p	0.02412	0.98546
hsa-miR-32-5p	0.02544	0.98546
hsa-miR-486-5p	0.02989	0.98546
hsa-miR-142-5p	0.04400	0.98546
hsa-miR-320b	0.04421	0.98546
hsa-miR-145-5p	0.04545	0.98546
hsa-miR-221-3p	0.04598	0.98546
hsa-miR-106a-5p	0.04701	0.98546

The table shows the 28 statistically significant miRNAs values with altered expressed levels among 100 miRNAs investigated during the observation time before correction for multiple testing and after correction (*n* = 8), sorted according to the lowest adjusted *p*-value.

**Table 3:** Enriched pathways associated with the validated target genes of the eight miRNAs with altered expression levels during the first five years after diagnosis.

Panther Pathways	Fold Enrichment	*p*-Value	miRNAs
Angiogenesis	3.37	8.25 × 10^−6^	hsa-miR-10b-5p, hsa-miR-125b-5p, hsa-miR-17-5p, hsa-miR-423-3p, hsa-miR-93-5p, hsa-miR-99a-5p
Gonadotropin-releasing hormone receptor pathway	2.9	1.77 × 10^−5^	hsa-miR-17-5p, hsa-miR-125b-5p, hsa-miR-497-5p, hsa-miR-423-3p, hsa-miR-10b-5p, hsa-miR-93-5p, hsa-miR-30e-5p, hsa-miR-99a-5p
TGF-β signaling pathway	4.08	7.21 × 10^−5^	hsa-miR-10b-5p, hsa-miR-125b-5p, hsa-miR-17-5p, hsa-miR-423-3p, hsa-miR-93-5p, hsa-miR-99a-5p
CCKR signaling map	3.11	1.07 × 10^−4^	hsa-miR-17-5p, hsa-miR-125b-5p, hsa-miR-497-5p, hsa-miR-93-5p, hsa-miR-99a-5p, hsa-miR-10b-5p, hsa-miR-423-3p
EGF receptor signaling pathway	3.12	1.16 × 10^−3^	hsa-miR-10b-5p, hsa-miR-125b-5p, hsa-miR-17-5p, hsa-miR-423-3p, hsa-miR-497-5p, hsa-miR-93-5p, hsa-miR-99a-5p
PDGF signaling pathway	2.91	3.16 × 10^−3^	hsa-miR-10b-5p, hsa-miR-125b-5p, hsa-miR-17-5p, hsa-miR-423-3p, hsa-miR-497-5p, hsa-miR-93-5p, hsa-miR-99a-5p
Apoptosis signaling pathway	3.12	5.05 × 10^−3^	hsa-miR-10b-5p, hsa-miR-125b-5p, hsa-miR-17-5p, hsa-miR-30e-5p, hsa-miR-423-3p, hsa-miR-93-5p
FGF signaling pathway	3.05	6.90 × 10^−3^	hsa-miR-10b-5p, hsa-miR-125b-5p, hsa-miR-17-5p, hsa-miR-423-3p, hsa-miR-497-5p, hsa-miR-93-5p, hsa-miR-99a-5p
p53 pathway feedback loops 2	4.49	7.85 × 10^−3^	hsa-miR-10b-5p, hsa-miR-125b-5p, hsa-miR-17-5p, hsa-miR-423-3p, hsa-miR-93-5p, hsa-miR-99a-5p
Ras Pathway	3.66	1.34 × 10^−2^	hsa-miR-10b-5p, hsa-miR-125b-5p, hsa-miR-17-5p, hsa-miR-423-3p, hsa-miR-497-5p, hsa-miR-93-5p
Integrin signalling pathway	2.41	3.07 × 10^−2^	hsa-miR-10b-5p, hsa-miR-125b-5p, hsa-miR-17-5p, hsa-miR-423-3p, hsa-miR-497-5p, hsa-miR-93-5p, hsa-miR-99a-5p

The enriched pathways are listed for the validated targets of the eight candidate miRNAs which changed in expression level over time. The fold enrichment and *p*-values were calculated using the binomial test. The *p*-values were adjusted for multiple correction using the Bonferroni method. TGF: transforming growth factor. CCKR: cholecystokinin receptors 1 and 2. EGF: epidermal growth factor. PDGF: platelet-derived growth factor. FGF: fibroblast growth factor. IGF: insulin-like growth factor.

**Table 4:** Association between the eight candidate miRNAs and autoantibodies titers.

Months after Diagnosis	miRNA	Autoantibody	Spearman Correlation (r_s_)	Unadjusted *p*-Value	Adjusted *p*-Values
3	hsa-miR-99a-5p	ICA	−0.35	0.036	N/S
3	hsa-miR-125b-5p	ICA	−0.36	0.030	N/S
3	hsa-miR-125b-5p	IA-2A	−0.35	0.032	N/S
6	hsa-miR-17-5p	GADA	0.37	0.033	N/S

The association between the eight candidate miRNAs and autoantibody titers measured at the five time points after diagnosis by Spearman correlation (r_s_). Only the statistically significant associations are shown (*p* ≤ 0.05). The significant values are before correcting for multiple testing. After correction, no significant association was seen (N/S). GAD65A: autoantibodies against antigen GAD. IA-2A: islet antigen 2. ICA: Islet-cell antibody. ZnT8: zinc transporter 8 (triple mix antibodies containing the isotypes glutamine, arginine, and tryptophan).

## Supplementary Materials

The following are available online at http://www.mdpi.com/2311-553X/4/4/35/s1, Table S1: ∆Cp values of 8 miRNAs at different time-points and Table S2: Pathway-based enrichment analysis using PantherDB.
