# Supplementary material for: Influence of Disease Duration on Circulating Levels of miRNAs in Children and Adolescents with New Onset Type 1 Diabetes
Source: Noncoding RNA. 2018 Nov 21;4(4):35. doi: 10.3390/ncrna4040035 (PMC6316625; doi:10.3390/ncrna4040035)
Supplement: Supplementary file 1 [file ncrna-04-00035-s001.zip › Table S1.docx]

**Supplementary Table S1.** ∆Cp values of eight miRNAs at different time-points

| **miRNA** | **1 month** | **3 months** | **6 months** | **12 months** | **60 months** |
| --- | --- | --- | --- | --- | --- |
| hsa-miR-93-5p | 2.26 | 2.05 | 2.07 | 2.1 | 2.33 |
| hsa-miR-30e-5p | 1.08 | 1 | 0.99 | 0.99 | 1.38 |
| hsa-miR-423-3p | 0.34 | 0.3 | 0.28 | 0.07 | 0.92 |
| hsa-miR-17-5p | -3.38 | -3.52 | -3.53 | -3.37 | -3.18 |
| hsa-miR-10b-5p | -3.56 | -3.25 | -3.06 | -3.37 | -4.4 |
| hsa-miR-99a-5p | -2.26 | -2.03 | -1.98 | -1.87 | -2.57 |
| hsa-miR-497-5p | -2.68 | -2.51 | -2.36 | -2.37 | -3.26 |
| hsa-miR-125b-5p | -2.76 | -2.58 | -2.41 | -2.36 | -3.16 |
